# Effectiveness of Virtual Reality Technology Interventions in Improving the Social Skills of Children and Adolescents With Autism: Systematic Review

**DOI:** 10.2196/60845

**Published:** 2025-02-05

**Authors:** Xipeng Yang, Jinlong Wu, Yudan Ma, Jingxuan Yu, Hong Cao, Aihua Zeng, Rui Fu, Yucheng Tang, Zhanbing Ren

**Affiliations:** 1 College of Physical Education Shenzhen University Shenzhen China; 2 College of Physical Education Southwest University Chongqing China; 3 School of Public Teaching Shanwei Institute of Technology Shanwei China; 4 Department of Education Shenzhen University Shenzhen China

**Keywords:** VR technology, autism spectrum disorder, children, adolescents, social skills, virtual reality, VR

## Abstract

**Background:**

Virtual reality (VR) technology has shown significant potential in improving the social skills of children and adolescents with autism spectrum disorder (ASD).

**Objective:**

This study aimed to systematically review the evidence supporting the effectiveness of VR technology in improving the social skills of children and adolescents with ASD.

**Methods:**

The search for eligible studies encompassed 4 databases: PubMed, Web of Science, IEEE, and Scopus. Two (XY and JW) researchers independently assessed the extracted studies according to predefined criteria for inclusion and exclusion. These researchers also independently extracted information regarding gathered data on the sources, samples, measurement methods, primary results, and data related to the main results of the studies that met the inclusion criteria. The quality of the studies was further evaluated using the Physiotherapy Evidence Database scale.

**Results:**

This review analyzed 14 studies on using VR technology interventions to improve social skills in children and adolescents with ASD. Our findings indicate that VR interventions have a positive effect on improving social skills in children and adolescents with ASD. Compared with individuals with low-functioning autism (LFA), those with high-functioning autism (HFA) benefited more from the intervention. The duration and frequency of the intervention may also influence its effectiveness. In addition, immersive VR is more suitable for training complex skills in individuals with HFA. At the same time, nonimmersive VR stands out in terms of lower cost and flexibility, making it more appropriate for basic skill interventions for people with LFA. Finally, while VR technology positively enhances social skills, some studies have reported potential adverse side effects. According to the quality assessment using the Physiotherapy Evidence Database scale, of the 14 studies, 6 (43%) were classified as high quality, 4 (29%) as moderate quality, and 4 (29%) as low quality.

**Conclusions:**

This systematic review found that VR technology interventions positively impact social skills in children and adolescents with ASD, with particularly significant effects on the enhancement of complex social skills in individuals with HFA. For children and adolescents with LFA, progress was mainly observed in basic skills. Immersive VR interventions are more suitable for the development of complex skills. At the same time, nonimmersive VR, due to its lower cost and greater flexibility, also holds potential for application in specific contexts. However, the use of VR technology may lead to side effects such as dizziness, eye fatigue, and sensory overload, particularly in immersive settings. These potential issues should be carefully addressed in intervention designs to ensure user comfort and safety. Future research should focus on optimizing individualized interventions and further exploring the long-term effects of VR interventions.

**Trial Registration:**

International Platform of Registered Systematic Review and Meta-analysis Protocols INPLASY202420079U1; https://inplasy.com/inplasy-2024-2-0079/

## Introduction

### Background

Autism spectrum disorder (ASD) is a neurodevelopmental condition marked by challenges in social interaction and communication, as well as by the presence of repetitive and stereotyped interests and behaviors [1]. The precise etiology of ASD remains elusive; however, studies indicate that a combination of genetic, environmental, and neurodevelopmental factors may contribute to its development [2]. Statistics from the US Centers for Disease Control and Prevention have shown a marked rise in the prevalence of ASD diagnoses in recent decades, with 1 in 77 children currently being diagnosed [3]. Social impairment is often characterized by difficulty interpreting social cues, including changes in facial expressions, body language, and tone of voice. This can lead to children and adolescents with ASD displaying a reduced interest in, or maladaptive responses to, social situations [4]. Currently, early intervention is an effective way to improve the social skills of children and adolescents with ASD [5,6].

Individuals with ASD experience significant deficits in social skills. These deficits manifest as difficulties in understanding others’ emotions and intentions, establishing and maintaining social relationships, and lacking flexibility in social interactions [7]. Such challenges often lead to increased social isolation and a decreased quality of life for those with ASD [8]. The severity of ASD symptoms varies; individuals with high-functioning autism (HFA) usually experience milder social impairments, while those with low-functioning autism (LFA) face more severe communication challenges [9]. Various interventions have been developed to address social skill deficits in individuals with ASD, achieving varying levels of success. For example, applied behavior analysis (ABA) is a therapeutic approach designed to improve social skill acquisition and enhance social motivation among individuals with ASD. Research by Roane et al [10] suggests that individualized, ABA-based interventions can significantly improve developmental outcomes for individuals with ASD, leading to progressive enhancements in social skills [11]. In addition, speech therapy offers personalized language and communication support, helping individuals gradually improve social communication skills. In the Social Communication Intervention Project (SCIP) led by Adams et al [12], it was found that speech therapy could enhance expressive language and comprehension, enabling individuals to gradually improve social skills by practicing conversations, learning communication techniques, and using assistive tools [13]. Cognitive behavioral therapy (CBT) is also widely used to address critical symptoms and socio-emotional challenges in children and adolescents with ASD. A meta-analysis by Wang et al [14] revealed significant improvements in both autistic symptoms and socio-emotional issues following CBT. Despite the considerable benefits of these traditional interventions, they have certain limitations. The high costs and the need for specific therapeutic environments and specialized therapists make these services inaccessible for many families, limiting their reach and frequency [15,16]. In addition, the lack of personalization in traditional methods may impact their effectiveness, especially for individuals with varying needs and responses [17]. These limitations underscore the importance of exploring alternative, more adaptable, individualized, and cost-effective intervention strategies.

Virtual reality (VR) technology, as a promising tool for promoting health, has gradually been applied in various fields, including gaming, education, health care, and psychotherapy. In gaming and entertainment, VR technology provides users with a highly immersive experience, allowing them to be fully immersed in a virtual world, which enhances interactivity and engagement [18,19]. VR simulates laboratories and historical scenes in education, enabling students to engage in hands-on learning within a safe virtual environment, thereby promoting deeper understanding and knowledge retention [20]. VR has been widely used in health care for rehabilitation, pain management, and psychotherapy [21,22]. Research by Rizzo and Koenig [23] suggests that VR technology provides a highly personalized interactive environment for psychotherapy and cognitive rehabilitation, helping patients better engage in treatment. Moreover, VR aids motor rehabilitation, particularly for patients with Parkinson disease and children with balance disorders, significantly enhancing recovery outcomes [24]. VR interventions can be categorized into immersive and nonimmersive types, depending on the realism of the simulated scenes [25]. Immersive VR typically uses head-mounted displays to fully engage the user’s senses, offering a highly realistic experience, thus making it particularly suitable for interventions requiring deep involvement [26]. In contrast, nonimmersive VR is experienced through traditional computer displays, offering greater flexibility and accessibility, thus making it more appropriate for everyday educational settings [27]. For ASD interventions, Parsons and Cobb [28] conducted an in-depth study on the application of VR technology, noting that VR can provide a safe virtual environment for children with ASD to practice social skills without real-world pressures. Therefore, VR technology holds significant potential as an innovative and adaptable intervention for addressing social skill deficits in children and adolescents with ASD.

### Objective

Although VR technology has shown great potential in the fields of education and therapy, especially in improving social skills in children and adolescents with ASD, empirical research remains limited. Therefore, this systematic review aims to evaluate the effects of VR interventions in ASD treatment and address the following key research questions: (1) What are the differences in the positive effects of VR interventions on the social skills of children and adolescents with HFA versus LFA? (2) How do different types of VR interventions impact the social skills of children and adolescents with ASD? and (3) Are there potential adverse effects of VR interventions on children and adolescents with ASD?

To answer these questions, this study aims to achieve the following objectives: (1) to compare the positive effects of VR interventions on the social skills of children and adolescents with HFA and LFA, (2) to analyze the impact of different types of VR interventions on the social skills of children and adolescents with ASD, and (3) to evaluate the potential adverse effects of VR interventions on children and adolescents with ASD.

## Methods

### Recruitment

Our research protocol was registered in the International Platform of Registered Systematic Review and Meta-analysis Protocols (ID: INPLASY202420079U1). This study followed the PRISMA (Preferred Reporting Items for Systematic Evaluation) guidelines to ensure transparency in the research process [29,30].

### Search Strategy

A comprehensive search was conducted across 4 electronic databases: PubMed, Web of Science, IEEE, and Scopus, as of April 11, 2024, to systematically identify pertinent studies concerning the impact of VR technology on improving the social abilities of children and adolescents with ASD. The search focused explicitly on peer-reviewed articles published in English. The search strategy incorporated keywords such as VR, ASD, social skills, communication skills, children, and adolescents. Multimedia Appendix 1 provides detailed information about the search terms used. Previous reviews have described the selection of search terms for VR interventions, social skills, and children and adolescents with ASD [31]. In addition, researchers manually searched the included studies’ reference lists to identify relevant articles.

### Eligibility Criteria

The criteria for inclusion in the study were developed using the PICOS (Population, Intervention, Comparison, Outcomes, and Study design) principles [32], as follows:

- Population: The participants were children and adolescents aged 3-18 years, diagnosed by a physician or by a reliable ASD assessment tool. Mixed studies on children and adolescents with attention deficit/hyperactivity disorder, Down syndrome, or other neurodevelopmental disorders were excluded.
- Intervention: This study investigated the implementation of VR technology as an intervention in clinical settings or clinical trials.
- Comparison: No restrictions were placed on the control group.
- Outcomes: All studies used a validated tool or methodology to assess the social competence of children and adolescents with ASD.
- Study design: There were no restrictions on trial design, including randomized and nonrandomized controlled trials.

### Study Selection and Data Extraction

Two researchers independently conducted a multistep search and assessed the studies based on the titles, abstracts, and full texts. To evaluate the risk of bias in the included studies, we used the Cochrane Risk of Bias tool [33]. This tool evaluates bias across several domains, including random sequence generation, allocation concealment, blinding of participants and personnel, blinding of outcome assessment, incomplete outcome data, selective reporting, and other sources of bias. Two (XY and JW) researchers independently assessed each study, resolving disagreements through discussion or consulting a third researcher [34].

The team created a data extraction form to collect information about each study’s characteristics, including details of the literature (authors and year), participant characteristics (sample size and age range), intervention components (location, intervention design, frequency, and duration), measurement instruments, and data related to primary outcomes. To increase transparency, we have included the PRISMA checklist in the Multimedia Appendix 2. This checklist provides a comprehensive overview of the methodology followed throughout the study, ensuring all relevant criteria are addressed and documented. This addition will allow readers to understand our research process better and replicate it.

### Quality Assessment

The methodological quality of each included study was independently assessed by 2 researchers according to the Physiotherapy Evidence Database (PEDro) scale [35]. The decision to use the PEDro scale was based on its widespread use and recognition in assessing the quality of clinical trials, particularly in physiotherapy and rehabilitation [35]. Despite the challenges posed by VR studies, the PEDro scale remains a valuable tool due to its comprehensive assessment of key methodological aspects, such as randomization and allocation concealment, which are critical for reducing bias in clinical research [36].

The PEDro scale consists of 11 scoring criteria: eligibility criteria (not scored), random allocation, concealed allocation, baseline comparability, blinding of subjects, blinding of therapists, blinding of assessors, sufficient follow-up, intention-to-treat analysis, between-group comparisons, and point estimates with variability [36]. PEDro scores ranged from 0 to 10, with a median score of 5. However, scoring individuals and therapists participating in VR technology trials can be challenging, and blinding participants or therapists in VR studies is often not feasible due to the nature of the technology, as both parties are usually aware of the ongoing intervention [37]. Therefore, considering the limitations of using VR technology, the scoring system can be categorized into 3 categories: high quality ≥6, appropriate quality=4-5, and low quality ≤3 [38]. In total, 2 (XY and JW) researchers independently assessed the methodological quality of the included studies according to the 11 PEDro criteria and calculated a total score to determine the quality of the study [39].

Disagreements regarding quality ratings should be deliberated until a consensus is achieved; if consensus proves elusive, a third researcher makes the final decision following a mutual discussion.

## Results

### Search Results

**Figure 1:**
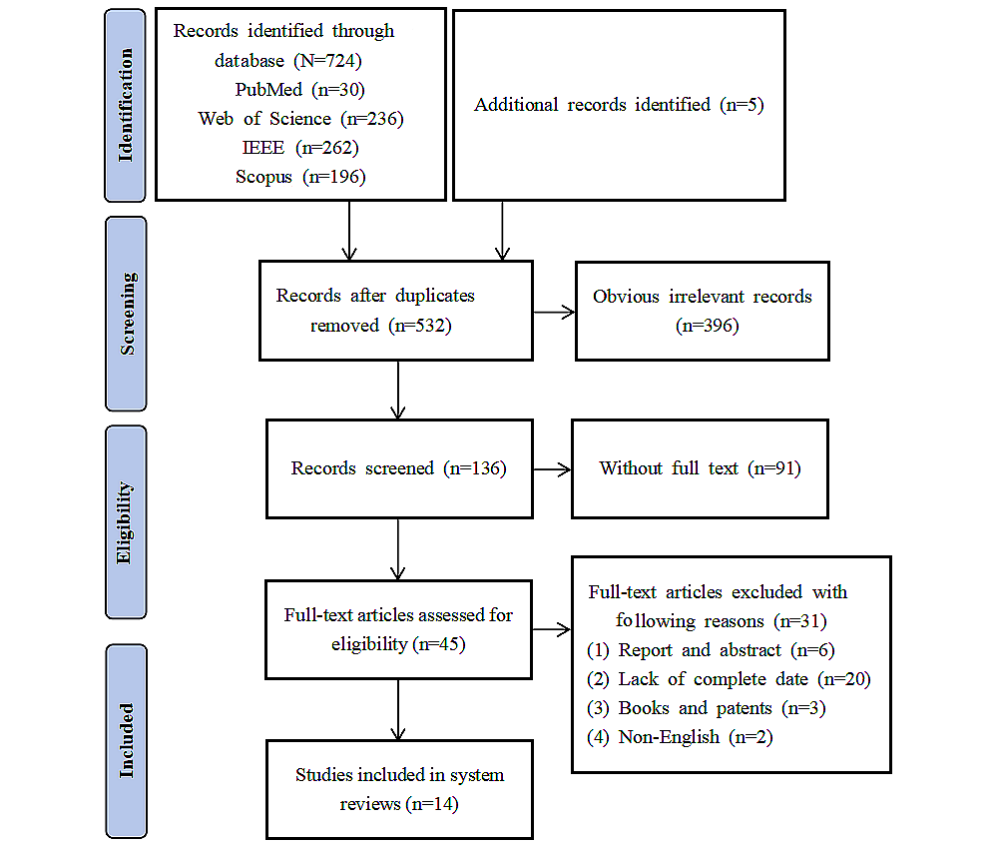
The flow chart of literature screening.

Figure 1 illustrates the specific search process, during which we initially retrieved 724 relevant studies: 30 from PubMed, 236 from Web of Science, 262 from IEEE, 196 from Scopus, and 5 additional studies (manual screening of reference lists and consultations with experts in the field). After excluding duplicates, 532 studies were included in the final analysis. First, 396 irrelevant studies were excluded based on title and abstract screening. The researchers removed 91 incomplete studies, leaving 45 studies for analysis. The final exclusion of reports and abstracts, studies lacking trial data, books, patents, and non-English language studies resulted in the inclusion of 14 studies.

### Research Characteristics

A total of 14 relevant studies were included in this review [40-53], with detailed characteristics of each study presented in Tables 1 and 2.

**Table 1:** Study characteristics.

Author	Country or region	Age range (year)	Diagnostic methods	Sample size, n (IG^a^ and CG^b^)	HFA^c^ or LFA^d^	Setting
Matsentidou and Poullis [40]	Cyprus	9-10	NR^e^	12	HFA	Clinical
Cheng et al [41]	Taiwan	10-13	NR	3	HFA	School
Ip et al [42]	Hong Kong	6-12	NR	72 (36 and 36)	HFA	School
Rosenfield et al [43]	United States	NR	NR	2	HFA	School
Junaidi et al [44]	Indonesia	13-18	NR	7	LFA	Clinical
Yeh and Meng [45]	Taiwan	7-14	NR	10	HFA	School
Tsai et al [46]	Taiwan	7-9	DSM-5^f^	3	HFA	Clinical
Soltani Kouhbanani et al [47]	Iran	6-12	DSM-5	30 (15 and 15)	HFA and LFA	Clinical
Moon and Ke [48]	United States	10-14	NR	15	HFA	School
Elkin et al [49]	United States	6-17	DSM-5	10	HFA and LFA	Clinical
Frolli et al [50]	Italy	9-10	DSM-5	60 (30 and 30)	HFA	Clinical
Ke et al [51]	United States	10-14	NR	7	HFA	Clinical
Zhao et al [52]	China	3-5	DSM-5	44 (22 and 22)	HFA and LFA	Clinical
Moon and Ke [53]	United States	12-13	DSM-5	4	HFA	Clinical

^a^IG: intervention group.

^b^CG: control group.

^c^HFA: high-functioning autism.

^d^LFA: low-functioning autism.

^e^NR: not reported.

fDSM-5: Diagnostic and Statistical Manual of Mental Disorders, Fifth Edition.

**Table 2:** Intervention information and main findings

References	Immersive or nonimmersive	Intervention	Frequency (length)	Type	Measures	Main findings
Matsentidou and Poullis [40]	Immersive	CAVE^a^	NR^b^	Chronic	Behavior observation	Results are “encouraging”
Cheng et al [41]	Immersive	3D-SU^c^	6 wk	Chronic	Social events card; social behaviors scale	Improved nonverbal communication, social initiations, and social cognition behaviors
Ip et al [42]	Immersive	CAVE	2/wk; 14 wk	Chronic	Psychoeducational Profile (Third Edition)	Social interactions (*P*＜.05); emotional expression (*P*＜.01)
Rosenfield et al [43]	Immersive	VR^d^	NR	Chronic	Behavior observation	To help effectively
Junaidi et al [44]	Nonimmersive	VR	NR	Acute	Behavior observation	Supports shopping skill development effectively
Yeh and Meng [45]	Nonimmersive	VR-SST^e^	1/wk; 8 wk	Chronic	Social skills evaluation sheet	Answer speed (*P*＜.05); conversation and expression (*P*＜.05); the completeness of sentence-structure (*P*＜.05); conversation etiquette (*P*＜.05)
Tsai et al [46]	Immersive	CAVE	5 wk	Chronic	Social Skills Training Scales	TPP-RPG^f^ system improved social skills (*P*<.01), maintained after intervention
Soltani Kouhbanani et al [47]	Nonimmersive	VR	7/wk; 12 wk	Chronic	Vineland Adaptive Behavior Scale	Social skills (*P*<.01); behavioral symptoms (*P*<.01)
Moon and Ke [48]	Nonimmersive	VR-SST	8 wk	Chronic	Jaccard Index	Treatment integrity scores improved (*P*<.05)
Elkin et al [49]	Immersive	VR-SC^g^	1 time	Chronic	Behavior observation	Enhances propensity for social interaction through gaze behavior analysis
Frolli et al [50]	Immersive	3D projection of primary emotions	3/wk; 12 wk	Chronic	Image testing	Primary emotions (*P*=.25); emotions and situations for primary emotions (*P*＜.05)
Ke et al [51]	Nonimmersive	VR-SST	2/wk; 15 wk	Chronic	Simulator Sickness Questionnaire; Social Communication Questionnaire	Simulator Sickness Questionnaire Self (*P*＞.05); Simulator Sickness Questionnaire Parent (*P*＞.05); Social Communication Questionnaire (*P*＞.05)
Zhao et al [52]	Nonimmersive	VR	3/wk; 12 wk	Chronic	Psychoeducational Profile (Third Edition)	Social interaction (*P*<.01); emotional expression (*P*＜.01)
Moon and Ke [53]	Nonimmersive	VR-SST	3/wk; 8 wk	Chronic	Simulator Sickness Questionnaire	Social-interaction initiation (*P*＜.05); responding to social interactions (*P*＞.05); interpersonal negotiation (*P*＜.05); self-identity (*P*＞.05); Simulator Sickness Questionnaire Self (*P*＞.05)

^a^CAVE: cave automatic virtual environment.

^b^NR: not reported.

^c^3D-SU: 3D Immersive Virtual Environment System to Enhance Social Understanding.

^d^VR: virtual reality.

^e^VR-SST: virtual reality social skills training.

^f^TPP-RPG: third-person perspective role-playing game.

^g^VR-SC: virtual reality safe course.

The 14 studies included 279 children and adolescents with ASD aged 3-18 years [40-53]. Six studies used the *Diagnostic and Statistical Manual of Mental Disorders, Fifth Edition* (DSM-5) to diagnose children and adolescents with ASD [46,47,49,50,52,53], while 8 studies had clinical diagnoses made by physicians (Table 1) [40,45,48,51].

Of these 14 studies, 5 were from the United States [43,48,49,51,53]; 1 from Hong Kong [42]; 1 from Iran [47]; 1 from Cyprus [40]; 1 from Indonesia [44]; 3 from Taiwan, China [41,45,46]; 1 from Italy [50]; and 1 from China [52]. In terms of the trial design, 1 study used a single case trial [44,50]; 4 used a randomized controlled trial [42,46,50,52]; and 9 used a single-group, before-and-after controlled trial [40,41,43,45,46,48,49,51,53] (Table 1).

Regarding trial sites, 9 studies were conducted in institutions or clinics [40,44,46,47,49-53], and 5 were conducted in homes or schools (Table 1) [41-43,45,48].

In terms of intervention modalities, 3 studies used a cave automatic virtual environment [40-42,46], 1 study used a 3D Immersive Virtual Environment System to Enhance Social Understanding [41], 4 studies used VR Social Skills Training [45,48,51,53], 1 study used VR SAFE Course [49], 1 study used a 3D projection of primary emotions [50], and 5 studies did not report the specific VR technology and content used (Table 2) [43,44,47,50,52].

The 14 studies had a minimum intervention period of 1 day [49] and a maximum of 15 weeks [51]. Notably, 3 studies did not report detailed information on the duration of the intervention (Table 2) [40,43,44].

To assess social competence, 2 studies used the Psychoeducational Profile (Third Edition) [42,52], 2 used the Simulator Sickness Questionnaire [51,53], 1 used the Social Events Card and Social Behaviors Scale [41], 1 used the Vineland Adaptive Behaviour Scale [47], and 1 used the Jaccard Index scores [48]. One study used image testing [50], 1 study used the Social Communication Questionnaire [51], 1 study used Social Skills Training scales and the Likert Scale [46], 1 study used speech data mining [53], 1 study used Social Skills Performance Evaluation Sheet [45], and 4 studies used behavioral observation and analysis (Table 2) [40,43,44,49].

### Quality Evaluation

Details of the quality assessments of the included studies are presented in Table 3. The 14 studies had an average PEDro quality (overall study quality) score of 5.21, of which 6 had an overall study quality score of ≥7. All 14 studies met the eligibility criteria and point measures criteria, reflecting a general consistency in study design and a general focus on outcome assessment. Random allocation, concealed allocation, and between-group comparison were implemented in 6 studies each. In contrast, a similar-at-baseline comparison was implemented in 10 studies, demonstrating that a proportion of the studies attempted to adopt the methodological criteria of randomization and control in their design to enhance the validity and credibility of the findings.

No studies implemented subject blinding, therapist blinding, or assessor blinding simultaneously, suggesting that the use of blinding was very limited in these studies, which may be due to the specific design and implementation settings of the studies. Most studies (n=11) reported a participant attrition dropout rate. Still, they did not perform intention-to-treat analyses, which may have affected the interpretation and generalization of the findings.

**Table 3:** Methodological quality assessment of included studies.

References	Eligibility criteria	Random allocation	Concealed allocation	Similar at baseline	Subject blinded	Therapist blinded	Assessor blinded	Dropout rate	Intention-to-Treat analysis	Between-group comparison	Point measures	Test of statistical significance	Overall study quality
Matsentidou and Poullis [40]	1^a^	0^b^	0	0	0	0	0	0	0	0	1	0	2
Cheng et al [41]	1	0	0	0	0	0	0	1	0	0	1	1	4
Ip et al [42]	1	1	1	1	0	0	1	1	0	1	1	1	9
Rosenfield et al [43]	1	0	0	0	0	0	0	0	0	0	1	0	2
Junaidi et al [44]	1	0	0	1	0	0	0	1	0	0	1	0	4
Yeh and Meng [45]	1	1	1	1	0	0	0	1	0	1	1	0	7
Tsai et al [46]	1	0	0	1	0	0	0	0	0	0	1	0	3
Soltani Kouhbanani et al [47]	1	0	0	0	0	0	0	0	0	0	1	0	2
Moon and Ke [48]	1	1	1	1	1	0	0	1	0	1	1	1	9
Elkin et al [49]	1	1	1	1	0	1	0	1	0	1	1	0	8
Frolli et al [50]	1	1	1	1	0	0	0	1	0	1	1	1	8
Ke et al [51]	1	1	1	1	0	0	0	1	0	1	1	0	7
Zhao et al [52]	1	0	0	1	0	0	0	1	0	0	1	0	4
Moon and Ke [53]	1	0	0	1	0	0	0	1	0	0	1	0	4

^a^1: Yes.

^b^0: No.

### Main Findings

#### Impact of VR Interventions on the Social Skills of Children and Adolescents With ASD

Among the 14 studies included [40-53], 71% (n=10) of the participants were individuals with HFA [40-43,45,46,48,50,51,53], 21% (n=3) included both participants with HFA and LFA [47,49,52], and 7% (1) involved only participants with LFA (Table 1) [44]. We found that VR interventions significantly improved the social skills of children and adolescents with HFA, while the improvements for participants with LFA were more limited (Table 4).

**Table 4:** The main results and conclusions of the included studies.

References	Main results	Adverse effects	Conclusions
Matsentidou and Poullis [40]	75% success in street crossing	Yes	VR^a^ improves social skills
Cheng et al [41]	Increases in initiation, cognition, non-verbal	NR^b^	3D-SU^c^ enhances social initiation skills
Ip et al [42]	Significant improvements in emotion and social interaction	Yes	VR enhances emotional regulation and social skills
Rosenfield et al [43]	Reduced response time in conversation	Yes	VR facilitates conversation practice
Junaidi et al [44]	Moderate effectiveness; high readability	Yes	VR moderately effective for clarity
Yeh and Meng [45]	Improved answer speed and conversation skills	NR	VR enhances conversation etiquette
Tsai et al [46]	Increased correct social responses	Yes	TPP-RPG^d^ improves social reciprocity
Soltani Kouhbanani et al [47]	Maintained social improvements at follow-up	NR	VR + risperidone enhances social behavior
Moon and Ke [48]	Enhanced initiation and negotiation skills	NR	Enhanced initiation and negotiation skills
Elkin et al [49]	Gaze behavior varies with ASD^e^ severity	NR	VR differentiates gaze behavior by severity
Frolli et al [50]	Faster emotion recognition	NR	VR accelerates social skills acquisition
Ke et al [51]	Improved social initiation and flexibility	Yes	VR enhances social interaction
Zhao et al [52]	Increased cognitive and social scores	NR	VR + rehab boosts cognitive or social skills
Moon and Ke [53]	Improved social interaction scores	NR	VR prompts improve social skills

^a^VR: virtual reality.

^b^NR: not reported.

^c^3D-SU: 3D Immersive Virtual Environment System to Enhance Social Understanding.

^d^TPP-RPG: Third-Person Perspective Role-Playing Game.

^e^ASD: autism spectrum disorder.

Studies focusing on HFA consistently demonstrated that VR social skills training systems significantly improved the social skills of children and adolescents with HFA [40-43,45-53]. Some studies also found that VR training effectively enhanced eye contact and the quality of social interactions in individuals with HFA [49]. Other research indicated that VR training helped HFA children and adolescents improve cognitive flexibility, social initiative, and conflict resolution skills [45,49,53]. In comparison, the effects of VR interventions on children and adolescents with LFA were less pronounced [40-53]. While VR-based shopping skills training was found to help individuals with LFA improve basic life skills to some extent, the overall progress was slower, and the degree of improvement was not as significant as in individuals with HFA [44]. Although some studies found that LFA participants did not perform as well as participants with HFA in VR settings, others still reported that VR interventions helped them progress in self-care skills [47,52]. Furthermore, the research emphasized that continuous improvements in adaptive prompts and personalized training could offer more support for individuals with LFA [52].

#### Impact of Different VR Intervention Approaches on the Social Skills of Children and Adolescents With ASD

Of the included studies, 64% (9/14) were conducted in institutional or clinical settings [40,44,46,47,49-53], while 36% (5/14) were conducted in-home or school environments (Table 1) [41-43,45,48]. We found that interventions conducted in institutional or clinical settings generally had greater control and professional oversight (Table 4) [40,44,46,47,49-53]. These settings often had specialized equipment and supervision by therapists, resulting in more significant intervention effects, which were better suited for highly customized interventions for children and adolescents with ASD [40,44,46]. However, the primary advantages of home- or school-based interventions were their convenience and real-world applicability [42,45]. Although these interventions had relatively less control, they still demonstrated benefits in terms of flexibility and cost-effectiveness [41-43,45,48].

Regarding intervention duration and frequency, 79% (11/14) of the studies reported this information [41,42,45-53], while 21% (3/14) did not [40,43,44]. The intervention duration ranged from as short as 1 day to as long as 15 weeks (Table 2) [49,51]. Compared to short-term interventions, we found that long-term interventions led to more significant improvements in social skills and cognitive flexibility (Table 4) [42,45-53]. In addition, higher-frequency interventions generally produced more noticeable improvements, while lower-frequency interventions, although beneficial, did not yield results as strong as high-frequency ones [52,53]. Lower-frequency interventions may not sufficiently reinforce the skills being taught, leading to weaker transfer effects [45]. Overall, the most effective intervention duration was found to be 6 to 15 weeks, with a frequency of at least 2-3 sessions per week [42,45-53]. This high-frequency, long-term intervention model provided participants ample opportunities to practice, ensuring effective social skills development and long-term retention [42,45-53].

Of the studies, 50% (7/14) used immersive VR technology [40-43,46,49,50], while 50% (7/14) used nonimmersive VR systems (Table 2) [44,45,47,48,51-53]. We found that immersive VR was more effective for complex social skills training, especially in participants with HFA (Table 4) [40-43,46,49,50]. Immersive VR technology, by providing rich multisensory stimulation, allowed participants to better grasp emotional expression and social interaction skills within simulated environments [40]. In more complex school scenarios, immersive VR better supports participants in practicing and transferring learned skills to real-life situations [42]. Finally, immersive VR training experiences received positive feedback, and no instances of motion sickness were reported [43]. While nonimmersive VR systems, such as desktop-based VR, did not offer fully immersive experiences, they still demonstrated advantages in terms of flexibility, cost, and adaptability [44,45,47,48,51-53].

#### Adverse Effects of VR Interventions on Children and Adolescents With ASD

Although VR technology significantly improved social skills, 6 studies reported adverse side effects associated with its use (Table 4) [40,42-44,46,51]. One study found that some children and adolescents with HFA experienced dizziness and nausea while using the cave automatic virtual environment system [40]. Another study reported that 71% (10/14) of children and adolescents with LFA experienced anxiety and fear when using head-mounted displays for the first time, and most participants felt fatigued after using VR devices [44]. In addition, 2 other studies also reported anxiety-related issues [46,51]. While overall, the use of VR technology for social skills training was positive, some children reported discomfort, although no cases of motion sickness were observed [43]. Another study indicated that children and adolescents with HFA experienced headaches and eye fatigue, particularly after prolonged use of VR devices [42].

## Discussion

### Principal Findings

This systematic review analyzed the effects of VR interventions on the social skills of children and adolescents with ASD. The results showed that VR interventions significantly impacted individuals with HFA, while the effects on those with LFA were more limited. The study also indicated that the duration and frequency of the interventions had a notable impact on their effectiveness. Moreover, immersive VR was found to be more suitable for training complex skills in individuals with HFA. At the same time, nonimmersive VR excelled in cost and flexibility, making it more appropriate for basic skill interventions in individuals with LFA. Finally, although VR interventions showed positive outcomes in improving social skills, some studies reported potential side effects, highlighting the need to account for individual differences when designing intervention programs and implementing measures to reduce adverse effects.

### Impact of VR Interventions on the Social Skills of Children and Adolescents With ASD

The differences in the effectiveness of VR interventions for children and adolescents with HFA and LFA can be attributed to variations in cognitive, social, and sensory processing skills [40-43,45,46,48,50,51,53]. Individuals with HFA generally have higher cognitive abilities and language skills, enabling them to understand better and adapt to complex social situations within VR environments [54]. This finding aligns with earlier studies that highlighted the need for more personalized intervention strategies based on ASD symptom severity [9-11]. These previous studies suggested that traditional interventions like ABA and CBT were more effective for high-functioning individuals due to their adaptability and cognitive skills, which is similarly observed in VR settings [9-11]. The advantages demonstrated by children and adolescents with HFA in VR interventions are partly due to their stronger abstract thinking skills and higher sensory tolerance [55]. Multisensory stimuli, such as the combination of visual, auditory, and tactile inputs, activate key brain regions, including the mirror neuron system and the prefrontal cortex, further enhancing social interaction and emotional understanding [56]. In addition, individuals with HFA are better at handling contextual changes within VR environments, adapting to feedback mechanisms in virtual scenarios, allowing them to quickly learn and transfer skills to real-life situations [42]. This adaptability explains why children with HFA generally exhibit faster progress in VR interventions. In contrast, individuals with LFA’s slower cognitive and language development hinders their progress in VR interventions [44,47,49,52]. This limitation reflects findings from traditional interventions, where children with LFA often progressed more in structured, basic tasks than in complex social or emotional skills [12,13,49]. Children with LFA often struggle with processing multisensory stimuli in virtual environments, making them prone to discomfort, such as sensory overload or anxiety reactions [44,57]. This sensory sensitivity confuses complex social scenarios, making it difficult for them to keep pace with the intervention [58]. Moreover, for individuals with LFA, cognitive and social difficulties limit their ability to master complex emotional expression or social interaction skills [49]. They tend to show progress in more structured, more straightforward tasks, such as life skills training or behavior management [44,52]. These limitations suggest that individuals with LFA require specialized support and simplified task designs to ensure gradual improvement in controlled settings.

Future interventions should be more personalized to bridge the gap in VR intervention outcomes between individuals with HFA and LFA [59]. For individuals with HFA, continued focus on complex social scenario training is essential. For individuals with LFA, interventions should emphasize managing sensory stimuli, reducing the risk of sensory overload, simplifying social interaction tasks, and offering more structured and incremental training content. The effectiveness of VR interventions is also significantly influenced by the setting in which they are conducted. Institutional and clinical environments generally offer specialized equipment and supervision by therapists, providing participants with highly customized support and real-time feedback [40,44,46,47,49-53]. These settings help ensure the quality and consistency of interventions, especially in emotional regulation and complex social skills training [60]. However, the limitations of institutional and clinical environments include high costs and limited scalability [40,47]. In contrast, home and school environments, although lacking immediate professional supervision, offer convenience and real-world applicability advantages, making them more suitable for skill transfer to everyday activities [41-43,45,48]. In addition, home and school settings reduce participants’ anxiety, as children are more relaxed and receptive to interventions in familiar environments [61]. The flexibility and accessibility of these environments make them suitable for long-term interventions, though they may compromise the depth and complexity of the intervention [61]. Future research should explore how to incorporate more professional support into home and school environments to ensure the effectiveness of interventions is not compromised.

### Impact of Different VR Intervention Approaches on the Social Skills of Children and Adolescents With ASD

The duration and frequency of interventions significantly impact the effectiveness of VR interventions. Longer intervention durations and higher frequencies generally have more significant and lasting effects, particularly in social skills and emotional regulation [41,46-48]. This observation is consistent with the findings in traditional therapies, where prolonged and frequent sessions in interventions like ABA and CBT have been linked to more sustainable outcomes in social skills improvement [11,14]. Shorter intervention durations or lower frequencies may not be sufficient to consolidate the skills learned, limiting the maintenance of intervention effects [42,45,49-53]. Long-term, high-frequency interventions provide more practice opportunities, helping participants gradually master and transfer these skills [62]. However, high-frequency and long-term interventions may also lead to fatigue, particularly in individuals with LFA who are more sensitive to sensory overload [44]. Therefore, future intervention designs should balance the duration and frequency of interventions, ensuring sufficient practice opportunities while avoiding sensory fatigue and overstimulation in participants. Further research should explore the combination of optimal intervention duration and frequency to ensure both effectiveness and sustainability.

The differences in the effectiveness of immersive and nonimmersive VR interventions stem from their varying levels of sensory stimulation and interaction methods [63]. Immersive VR, with its multisensory stimulation and highly realistic virtual environments, is particularly effective for children with HFA, enhancing their adaptability in complex social interactions [40-43,46,49,50]. However, its intense sensory input may pose challenges for individuals with LFA, potentially causing anxiety or sensory overload [44,45,47,48,51-53]. As a lower-stimulus alternative, nonimmersive VR supports adaptive training for children with LFA, focusing on basic skills and behavior management [64]. Its ease of use and scalability make it suitable for broader and long-term applications, particularly in educational settings where lower-stimulus environments are often preferable [15,17]. Future research should prioritize integrating the strengths of immersive and nonimmersive VR to balance engagement and usability, ensuring interventions can address the diverse needs of individuals with ASD effectively.

### Practical Implications and Adverse Effects

VR technology interventions hold significant potential for clinical and educational applications in children and adolescents with ASD [65]. In clinical practice, VR can serve as a complementary tool to traditional interventions, providing therapists with a flexible and engaging means to meet the diverse needs of individuals with ASD [66]. For instance, the benefits of VR as an adjunct to speech therapy align with findings from the SCIP intervention in traditional therapy, where tailored approaches proved beneficial for improving social communication skills in children with ASD [12]. For instance, traditional speech therapy interventions, such as SCIP, have been shown to effectively improve social communication skills in children with ASD [12]. Integrating VR into these interventions could enhance the effects of traditional speech therapy, offering more engaging and personalized treatment sessions [67]. In addition, a meta-analysis of CBT’s effectiveness in addressing social-emotional challenges in children with ASD has shown significant improvements in core symptoms following CBT [14]. By incorporating VR into CBT, children can practice coping strategies in simulated real-life emotional and social scenarios, potentially enhancing outcomes [68]. Finally, VR could be combined with ABA to create personalized social scenarios, allowing therapists to offer more dynamic and adaptable intervention plans while children practice social behaviors in realistic but controlled environments [11,69].

Despite the many advantages of VR interventions, there are challenges to its widespread application, particularly regarding accessibility, cost, and the training required for educators and therapists to implement these technologies [70,71] effectively. Previous studies on traditional therapy have also highlighted these issues, where high costs and professional training needs limit scalability [15,16]. Adequate training for therapists and educators is crucial to maximizing the potential of VR interventions [72]. Research has highlighted that while immersive VR offers powerful therapeutic effects, there are challenges related to user comfort and sensory overload, especially for children with ASD. It is essential to carefully manage symptoms such as dizziness, nausea, and sensory overload, emphasizing the importance of gradual exposure in VR-based interventions [73]. In addition, studies have reported adverse effects such as eye fatigue, headaches, anxiety, and disorientation during VR use [40,42-44,46,51]. These reactions are particularly concerning for children with ASD, who may exhibit heightened sensitivity to sensory stimuli [74]. For instance, immersive VR systems with complex visual and auditory elements have been found to increase the risk of dizziness and nausea in approximately 15% of users [75]. Such adverse effects may reduce engagement and limit VR interventions’ scalability [76]. To address these challenges, intervention designs must incorporate strategies to minimize adverse effects. These include shortening session durations, integrating regular breaks, and adapting VR content to reduce sensory overload [77]. For example, designing environments with simpler visual elements or offering less immersive options for highly sensitive children could significantly mitigate these effects [78]. Furthermore, providing psychological support during interventions, such as teaching coping mechanisms for anxiety, may enhance user comfort and overall feasibility [79]. Future research should also systematically evaluate the prevalence and impact of these adverse effects, offering data-driven recommendations for optimizing VR-based interventions.

Moving forward, research should focus on improving the affordability and user-friendliness of VR interventions, ensuring that they are not limited to professional settings but can be widely applied in both clinical and educational environments [80].

### Limitations

Despite the many strengths of this study, several limitations require further discussion. The sample size of this systematic review is relatively small, including only 14 studies, which may limit the generalizability of the findings. The included studies used various scales and tools to assess the social skills of children and adolescents with ASD, and this diversity in assessment tools may have led to inconsistencies in the pooled results. Due to differences in evaluation tools and reported data types, this study could not provide a quantitative summary. There was significant heterogeneity in intervention protocols, including variations in VR content, session length, and frequency. This lack of standardization limits comparisons between studies and highlights the need for uniform intervention protocols in future research. Many of the included studies had small sample sizes, which reduced statistical power and increased the likelihood of underestimating the effects of VR interventions. Future research should recruit larger and more diverse samples to enhance the reliability and generalizability of findings. Finally, some studies lacked detailed reporting of key methodological elements, such as randomization and blinding, which increases the potential for bias. Adopting rigorous designs and transparent reporting practices, guided by frameworks such as PRISMA or CONSORT (Consolidated Standards of Reporting Trials), will help strengthen the evidence base for future studies. By addressing these limitations, this study underscores the importance of more robust and standardized research to advance the field of VR interventions for ASD.

### Conclusion

This systematic review highlights the significant potential of VR technology interventions in enhancing social skills for children and adolescents with ASD (Textbox 1).

In conclusion, long-term, high-frequency VR interventions (especially those lasting 6 to 15 weeks with 2-3 sessions per week) demonstrate the most effectiveness. Future research should focus on optimizing VR interventions to meet the personalized needs of different subgroups of patients with ASD and conduct large-scale longitudinal studies to validate their long-term impact.

**Key insights on virtual reality (VR) interventions for children and adolescents with autism spectrum disorder (ASD).**

- Differences in VR intervention effects for individuals with high-functioning autism (HFA) and low-functioning autism (LFA): VR interventions show considerable improvements in complex emotional regulation and social interaction skills for children and adolescents with HFA. In contrast, improvements for individuals with LFA have been relatively limited, primarily supporting basic life skills. This suggests the need for more tailored VR approaches for different ASD subgroups.
- Impact of different VR intervention types: Immersive VR proves more effective for enhancing complex social skills, particularly in children with HFA, as it provides a deeply engaging experience. Meanwhile, due to its flexibility and lower cost, nonimmersive VR offers valuable benefits in specific contexts, particularly in home or school settings. Institutional and clinical settings have shown more specialized and effective results, whereas interventions in home or school environments provide greater adaptability.
- Potential adverse effects of VR interventions: Although VR technology holds promise, adverse effects such as dizziness and fatigue may arise, especially during immersive VR sessions. These potential issues should be carefully addressed in the design of VR-based interventions.
- Practical recommendations for stakeholders: Therapists, educators, and developers should collaborate to optimize VR interventions for children with ASD. Therapists can integrate VR as a complementary tool to traditional therapies, tailoring interventions and gradually increasing exposure to reduce sensory overload. Educators can use VR for social skills training in controlled environments with proper training. Developers should focus on creating cost-effective, user-friendly VR platforms with customizable features to ensure accessibility in clinical and educational settings. These efforts will enhance the scalability and impact of VR interventions.

## Data Availability

All data generated or analyzed during this study are included in this published paper (and Multimedia Appendices 1 and 2).

## Appendix

Multimedia Appendix 1 Search research strategy.

Multimedia Appendix 2 PRISMA (Preferred Reporting Items for Systematic Reviews and Meta-Analyses) checklist.

## Abbreviations

ABA: applied behavior analysis
ASD: autism spectrum disorder
CBT: cognitive behavioral therapy
CONSORT: Consolidated Standards of Reporting Trials
DSM-5: Diagnostic and Statistical Manual of Mental Disorders, Fifth Edition
HFA: high-functioning autism
LFA: low-functioning autism
PEDro: Physiotherapy Evidence Database
PICOS: Population, Intervention, Comparison, Outcomes, and Study design
PRISMA: Preferred Reporting Items for Systematic Evaluation
SCIP: Social Communication Intervention Project
VR: virtual reality
