# Supplementary material for: Effectiveness of Virtual Reality Technology Interventions in Improving the Social Skills of Children and Adolescents With Autism: Systematic Review
Source: J Med Internet Res. 2025 Feb 5;27:e60845. doi: 10.2196/60845 (PMC11840372; doi:10.2196/60845)
Supplement: Multimedia Appendix 1 [file jmir_v27i1e60845_app1.docx]

**Multimedia Appendix** **1:Search research strategy**

| Database | Search strategy | Limits |
| --- | --- | --- |
| PubMed:(By using title/abstract)  Web of science:(By using topic)  IEEE:(By using abstract)  Scopus:(By using title/abstract/key) | (“Virtual Reality” OR “VR” OR “Virtual Reality Intervention” OR “Immersive Virtual Reality” OR “Non-Immersive Virtual Reality” )  AND  (“Social skills” OR “Social abilities” OR “Social contact” OR “Interpersonal Skills” OR “Interpersonal Skill” OR “Social Competence”)  AND  (“Communication Disorder” OR “Communicative Disorders” OR “Communicative Disorder” OR “Communicative Dysfunction” OR “Communicative Dysfunctions” OR “Neurogenic Communication Disorders” OR “Neurogenic Communication Disorder” OR “Communication Disabilities” OR “Communication Disability” OR “Acquired Communication Disorders” OR “Acquired Communication Disorder”)  AND  (“Autism Spectrum Disorder” OR “Autism” OR “ASD” OR “Autistic” OR “Asperger Syndrome” OR “Pervasive Developmental Disorder” OR “PDD” OR “PDDNOS”)  AND  (“children” OR “childhood” OR “school-age” OR “youth” OR “adolescents” OR “teenagers” OR “students”) | -Human；-English language |
